# Broadband Dual-Polarized Base Station Antenna for Fifth-Generation (5G) Applications

**DOI:** 10.3390/s18082701

**Published:** 2018-08-17

**Authors:** Hua Tang, Xianzheng Zong, Zaiping Nie

**Affiliations:** School of Electronic Science and Engineering, University of Electronic Science and Technology of China, No.4, Section 2, North Jianshe Road, Chengdu 610054, China; zpnie@uestc.edu.cn

**Keywords:** 5G antenna, broadband, base station antenna, dual-polarized, high port isolation

## Abstract

A broadband dual-polarized base station antenna with special designed feeding structures is investigated in this paper. The proposed antenna contains two pairs of crossed dipoles, two specially designed feeding connectors, two pieces of dielectric pads, a supporter (also a balun), and a reflector. To verify the designed antenna, a prototype is fabricated and measured. The antenna attains a bandwidth of around 46.5% operating over 3.14–5.04 GHz under reflection coefficient lower than −15 dB, and the port-to-port isolation is higher than 32.5 dB. It also achieves very stable radiation patterns with half power beam widths of 71.8° ± 2.5° in both the horizontal and vertical planes and gains of around 8 dBi over its operating band. Besides, the mechanism of the obtained good performances is clearly explained from the angle of current. All of the features ensure that the proposed antenna is suitable for the fifth-generation (5G) mobile communications.

## 1. Introduction

To support the fast development of international mobile telecommunication system-2020 (IMT-2020), commonly known as the fifth-generation (5G) mobile communications, many areas and countries have licensed the possible frequency bands for researches and tests, including the sub-6 GHz bands and millimeter wave bands. In particular, the spectrum availability in the sub-6 GHz frequency ranges, i.e., 3.3–4.2 GHz and 4.4–5.0 GHz, is growing globally [1], say 3.3–3.6 GHz and 4.8–5.0 GHz in China, 3.4–3.8 GHz in Europe, and 3.4–3.7 GHz in Korea, etc.

Various base station antennas are proposed for the third-generation (3G) mobile communications and long-term evolution (LTE) in the past decades [2,3]. Though the antenna reported in [2] attains a broad impedance bandwidth, the criterion it referred to is voltage standing wave ration (VSWR) < 2, which is lower than the common requirement in applications. Besides, it suffers narrow half power beam widths (HPBWs), varying from 58° to 64.5°. The antenna presented in [3] provides good performances in general, yet its HPBWs vary from 58.1° to 72.6°, which means that the undulation range is a little bit large. Moreover, their minimum beam widths need to be improved to meet the common requirement in applications.

At present, however, most of the researches on 5G are mainly about beamforming [4], massive multiple-input multiple-output (MIMO) antenna selection [5,6], etc., while the study on base station antenna for 5G mobile communications has not been paid too much attention to. Moreover, many of design examples focus on the millimeter wave bands [7,8]. Only several studies pay attention to the base station antenna design for the sub-6 GHz 5G applications [9,10,11,12]. A stacked antenna [9] operating at 3.7 GHz with an absolute bandwidth of 150 MHz is proposed for 5G mobile communications, which attains the HPBW of around 53.5°. The narrow bandwidth and HPBW limit its application in 5G, especially its narrow bandwidth. A MIMO antenna operating over 3.4–3.8 GHz [10] is proposed for the sub-6 GHz 5G applications, while its port isolations and realized gains are very low, only 11.5 dB and 4.1 dBi. Another MIMO antenna covering 3.2–3.9 GHz is designed for 5G [11]. Unfortunately, the bandwidth is still too narrow to cover the whole sub-6 GHz band for 5G applications. The investigation in [12] reports a wideband microstrip antenna operating over 2.84–5.17 GHz under a reflection coefficient lower than −10 dB, whereas it suffers a comparatively lower peak gain, only 6.2 dBi. More importantly, it only gets a single polarization.

In this work, a broadband dual-polarized base station antenna with high port isolation and stable radiation pattern is proposed for 5G application. Detailed design of the proposed antenna is presented, as well as its prototype. The measurements of the prototype agree with the simulated results well on the whole. The proposed antenna achieves a bandwidth of about 46.5% over 3.14–5.04 GHz under a reflection coefficient lower than −15 dB, wider than those of in [9,10,11]. The minimum port-to-port isolation reaches 32.5 dB and HPBWs fall into 71.8° ± 2.5°, which are better than those of the antennas in [9,10,11,12]. In addition, the presented antenna attains the realized gains of around 8 dBi, which is obviously higher than that of in [12], over the operating band. A full performance comparison for the proposed antenna and other designs for the same applications are summarized in Table 1. Apparently, the performances of the proposed antenna not only are better than those of the existing antennas reported for sub-6 GHz 5G applications on the whole, but also guarantee its well application in 5G mobile communications.

The main contributions of this work can be summarized in the following aspects. The proposed antenna focuses on the 5G’s sub-6 GHz band which has not been paid enough attention to at present. Well-designed antenna structures, especially the special designed feeding structures, allow the proposed antenna to outperform other existing antennas for the same applications on the whole. Compared with other designs, the proposed antenna satisfies the common requirements of mobile communication well and is more suitable for 5G’s application over the sub-6 band. Moreover, the performances of the proposed antenna in bandwidth, port isolation, and HPBW are obviously better than other studies [9,10,11,12]. Besides, the mechanism of the good performances the antenna achieved is clearly explained from the angle of current in this paper.

The remainder of this paper is organized as follows. Section 2 presents the configuration of the proposed antenna with detailed geometrical information and the design considerations. The performances of the proposed antenna are fully examined in Section 3. The simulated and measured results are also provided in this section, including the related discussions. Conclusions are drawn in Section 4.

## 2. Antenna Structure

**Figure 1:**
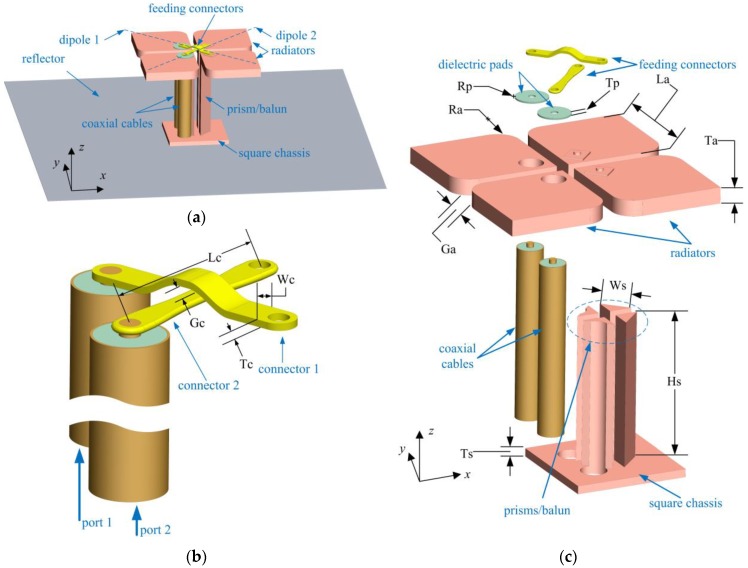
Proposed antenna configuration: (**a**) overview of the antenna, (**b**) detailed view of feeding structure, and (**c**) assembly view of the antenna structure without reflector.

The configuration of the proposed broadband dual-polarized base station antenna is depicted in Figure 1a, where the antenna consists of two pairs of crossed dipoles, two pieces of feeding connectors, two pieces of dielectric pads, a supporter (also a balun) with a height of Hs = 18 mm, and a reflector with a dimension of 80 mm × 80 mm. The proposed antenna is supported by four prisms which are united by a square chassis with a thickness of Ts = 1 mm at the bottom. This supporter also plays the role of balun, whose height is around 0.25λ_0_ (λ_0_ is the central wavelength) in this design. Each pair of the dipoles is excited by a coaxial cable that passes through a hole in the reflector and the chassis of the supporter. One arm of a dipole is connected to the outer conductor of the coaxial cable, and the other is connected to the inner conductor of the coaxial cable via a feeding connector. Two pieces of poly tetra fluoroethylene pads with a dielectric constant of *ε_r_* = 2.2 are installed as separators voiding the contacts between the feeding connectors and the radiators who are connected to the outer conductors of the coaxial cables. The two pads are also used to support the feeding connectors. Detailed feeding configuration and antenna structure are clearly depicted in Figure 1b,c.

**Figure 2:**
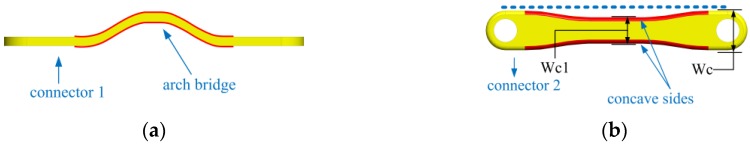
The detailed view of feeding connectors: (**a**) connector 1 in side view and (**b**) connector 2 in top view.

For the two crossed dipoles, the crossed feeding connectors usually result in a difference in the length of current path. In order to gain the same length of current path for the two crossed dipoles, the feeding connectors are carefully designed, as illustrated in Figure 2. Feeding connector 1 is shaped like an arch bridge, which is marked in red in Figure 2a, to cross over feeding connector 2. However, when the current flows through this feeding connector, the length of the current path is increased, compared with a straight feeding structure. This is because the arch structure results in an extra current path. In order to increase the length of the effective current path for the port 2 and offset the extra current length caused by the arch of connector 1, feeding connector 2 is consciously designed with a concave at both sides, as marked in red in Figure 2b. When port 2 is excited, the current flows from one radiator to another radiator via the edges of the connector 2. Accordingly, due to the designed concaves at both sides of connector 2, the current path on connector 2 is altered and an extra length of the current path is thereby obtained. The concave design on the sides of connector 2 can be regarded as a current path compensation. As a result, by tuning the value of Wc1 properly and carefully, both ports can obtain the same length of effective current path. This design also guarantees the symmetry of the current distribution of the dipoles excited by port 1 and port 2.

**Figure 3:**
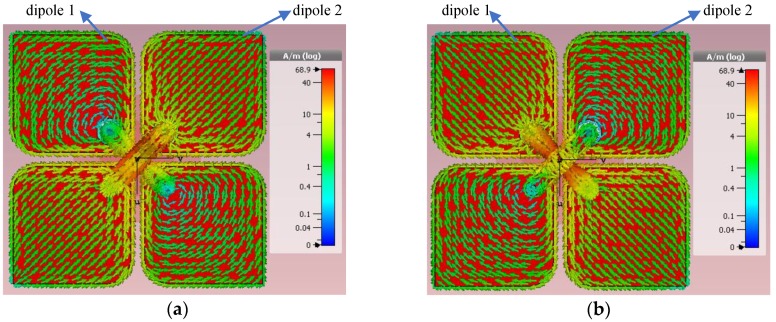

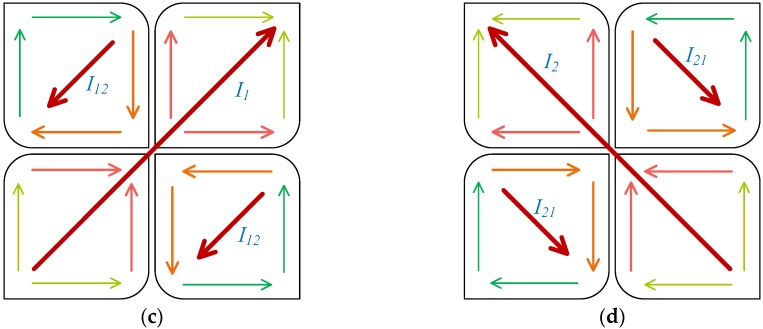
Current distribution excited by (**a**) port 1 and (**b**) port 2, and the equivalent current on the radiators corresponding to the excitations of (**c**) port 1 and (**d**) port 2 at a phase of π/4.

An observation of current distribution is illustrated in Figure 3a,b where the current phase is π/4. It can be found from Figure 3a that the current distribution on the radiators of dipole 2 is highly symmetrical with regard to the axis of dipole 1. A similar current distribution can be seen in Figure 3b. Since the radiators of dipole 1 and dipole 2 are the parasitic elements to each other, when dipole 1 is excited, the current distributed on dipole 2 is the introduced current caused by mutual coupling, and vice versa. The equivalent current illustrations corresponding to Figure 3a,b are presented in Figure 3c,d, respectively. For the excitation of port 1, the current on dipole 1 is equivalent to *I*_1_ and the current on dipole 2 introduced by mutual coupling is equivalent to *I*_12_; as to the excitation of port 2, the current on dipole 2 is equivalent to *I*_2_ and the current on dipole 1 introduced by mutual coupling is equivalent to *I*_21_. Since *I*_1_ and *I*_12_ generate the lower band and higher band resonances for dipole 1, and *I*_2_ and *I*_21_ generate the lower band and higher band resonances for dipole 2, respectively, the proposed antenna achieves a broad impedance bandwidth. This can be found in the simulated and measured S11 and S22 in Figure 5. Furthermore, it can be found that the directions of the equivalent current corresponding to the excitation of port 1 are perpendicular to the ones corresponding to the excitation of port 2, that is, the directions of *I*_1_ and *I*_12_ are always orthogonal to those of *I*_2_ and *I*_21_. As, for a dipole antenna, the direction of linear polarization is same as that of its exciting current, the linear polarizations excited by *I*_1_ and *I*_12_ are always perpendicular to those excited by *I*_2_ and *I*_21_. Accordingly, the proposed antenna obtains two orthogonal polarizations under both ports being excited. Moreover, the highly symmetrical distributions of the introduced current ensure that the current flowing between the two parasitic radiators, e.g., radiators of dipole 2 when dipole 1 being excited, is very small. It means that the current flowing between two ports is small enough. As a consequence, high port-to-port isolation is attained by the proposed antenna.

The detailed geometrical parameters of the proposed broadband dual-polarized antenna are summarized in Table 2.

## 3. Results and Discussion

**Figure 4:**
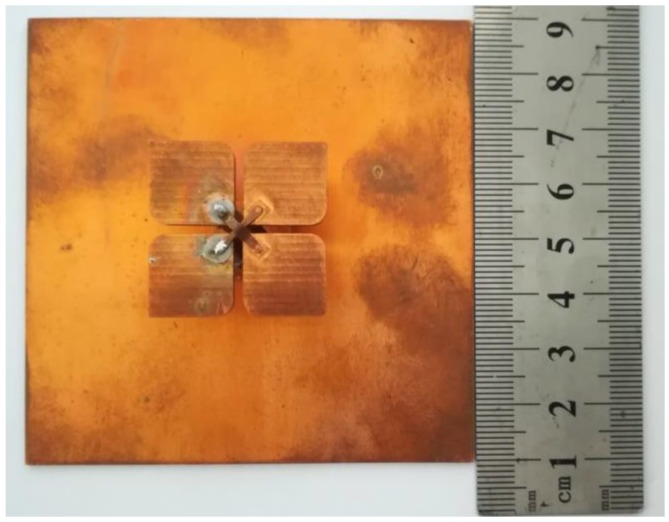
Photo of the proposed antenna prototype.

To verify our design, a prototype of the antenna is fabricated, as shown in Figure 4, and the simulated and measured results are presented to show the performances of the proposed broadband dual-polarized antenna in this section. Due to the manufacture precision, the dimensions of the prototype cannot be exactly same as those of the simulation model, especially those of the feeding connectors, which directly affect the antenna’s input impedance. Consequently, some differences occur between the performances of the measured and simulated results. In general, the measured results show high agreement with the simulated ones.

**Figure 5:**
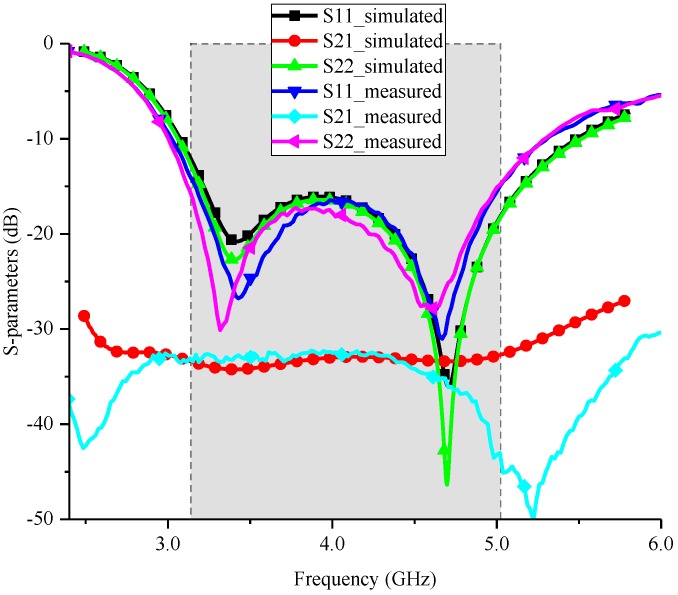
Measured and simulated S-parameters of the proposed antenna.

The measured and simulated S-parameters are presented in Figure 5. The results show that the measured S11 and S22 agree with the simulated ones well in general, except for a small frequency shift toward lower frequency. Moreover, although there is a small shift, the measured S11 and S22 still fully cover the required frequency range well, namely S11 < −15 dB and S22 < −15 dB in the frequency range of 3.14–5.04 GHz. In addition, a high agreement in reflection coefficient can be found at two ports. This agreement is mainly benefited from the special design of the two feeding connectors which lead to the same effective length of current path for the two crossed dipoles. Besides, the measured port-to-port isolation, i.e., S21, shows that the proposed antenna can provide a port isolation of at least 32.5 dB over its working frequency band. The high port isolation can be obtained by the proposed antenna, because the introduced current distributed on the two radiators of a dipole is highly symmetrical with regard to the axis of the other dipole, as illustrated in Figure 3. Therefore, the introduced current flowing from one radiator of a dipole towards another radiator is very small, when the other dipole is excited. In other words, the introduced current in port 1 is very small when port 2 is excited, and vice versa. This kind of balance can be guaranteed by the well-designed balun structure and feeding connectors.

**Figure 6:**
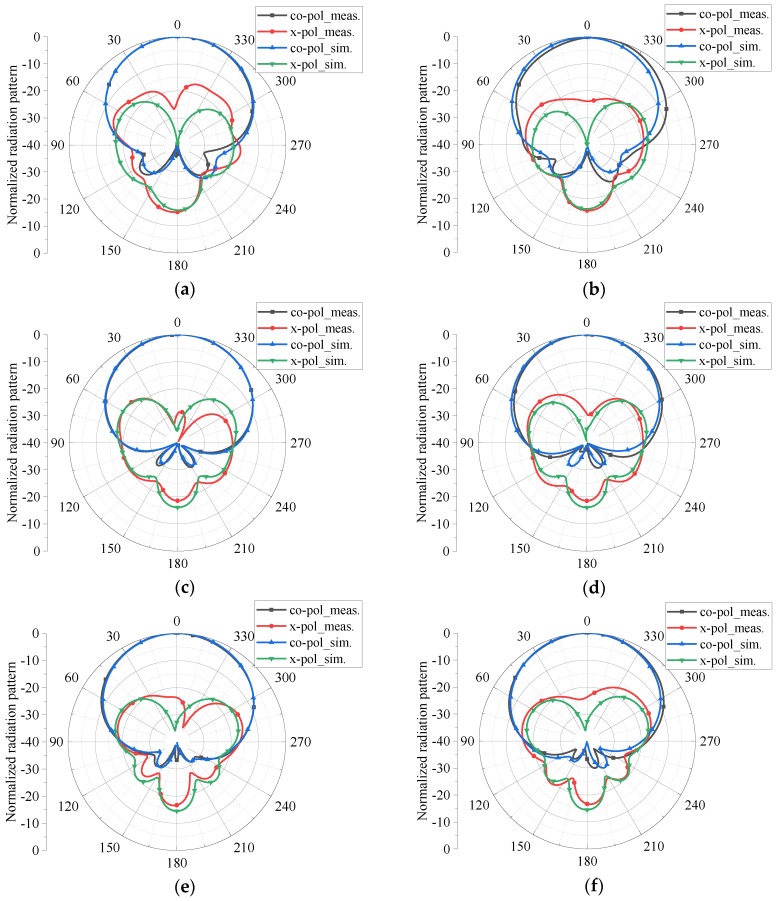
Normalized radiation patterns of the proposed antenna in H-plane at (**a**) 3.3 GHz, (**c**) 4.15 GHz, and (**e**) 5.0 GHz, and in V-plane at (**b**) 3.3 GHz, (**d**) 4.15 GHz, and (**f**) 5.0 GHz, excited by port 1.

The measured and simulated normalized radiation patterns in horizontal plane (H-plane) and vertical plane (V-plane), namely *xz*-plane and *yz*-plane shown in Figure 1, for the excitation of port 1 at 3.3 GHz, 4.15 GHz, and 5.0 GHz are plotted in Figure 6, where “co-pol” and “x-pol” in the labels represent the co-polarization and cross-polarization and the suffixes, “meas.” and “sim.”, stand for the measured and simulated results, respectively. It can be seen that the co-polarization components of the measured normalized radiation patterns are highly consistent with the simulated ones for all observation frequencies. Though there are some differences between the cross-polarization components of the measured and simulated radiation patterns, the measured results are on the whole coincident with the simulated ones. In addition, the results suggest that high agreements in radiation patterns in H-plane and V-plane are obtained across its operating band.

The radiation components of co-polarization and cross-polarization for port 1 describe the radiation of the linear polarization generated by dipole 1 with the direction along the axis of dipole 1. As to the radiation of the other orthogonal linear polarization generated by dipole 2 with the direction along the axis of dipole 2, it also can be decomposed into co-polarization and cross-polarization components. Since the structure of the antenna is highly symmetrical, the similar radiation patterns can be obtained when the antenna is excited by port 2. Accordingly, the radiation patterns excited by port 2, i.e., the radiation of the other orthogonal linear polarization, are not presented.

The results of measured HPBWs in H-plane and V-plane for the excitation of port 1 are recorded in Table 3. It can be found that the HPBWs of the antenna in H-plane and V-plane vary from 69.3° to 73.2° and from 70.9° to 74.3°, respectively. It implies that the proposed antenna attains very stable radiation patterns with HPBWs varying in 71.8° ± 2.5° over the operating band, which can also be seen in Figure 6.

**Figure 7:**
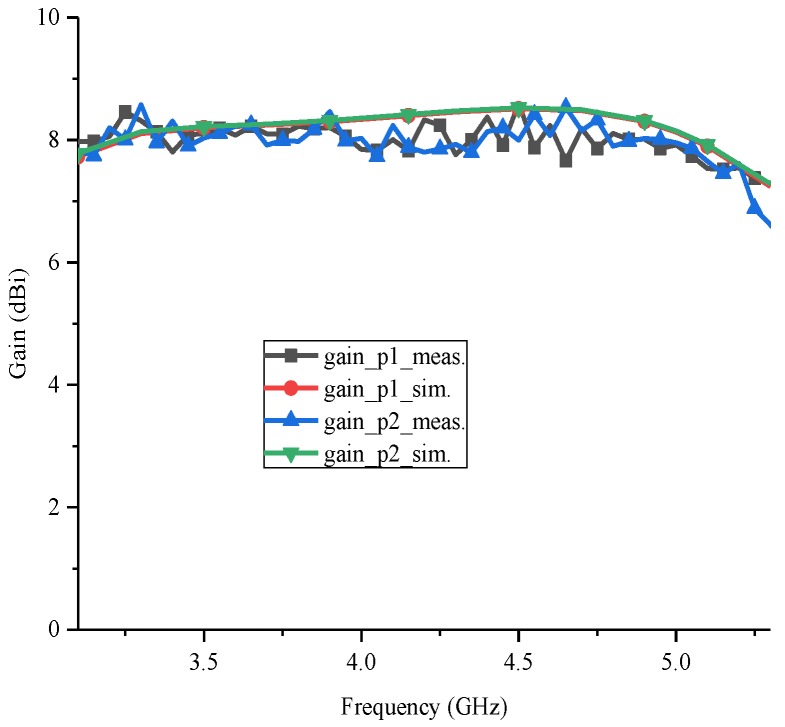
Measured and simulated gains of the proposed antenna.

Figure 7 presents the measured and simulated gains of the proposed antenna. Though the measured gains are a little lower than the simulated ones, and the measured results suffer a very small fluctuation, around ±0.3 dB, the measured results are on the whole coincident with the simulated ones for the excitations at both ports, obtaining gains of around 8 dBi. It suggests that the proposed antenna can provide stable gains over the working band.

## 4. Conclusions

In this paper, an antenna with a special designed feeding structure is proposed for 5G mobile communications in sub-6 GHz bands. The characteristics of the antenna are fully demonstrated by the measurements which agree with the simulated ones well on the whole. According to the measured results, the proposed antenna achieves a reflection coefficient lower than −15 dB and a minimum port-to-port isolation higher than 32.5 dB over 3.14–5.04 GHz, obtaining a bandwidth of 46.5%. Moreover, the antenna shows very stable radiation patterns with HPBWs varying in 71.8° ± 2.5° and average gain of around 8 dBi. In general, the presented antenna outperforms the existing antennas proposed for sub-6 GHz 5G applications. Therefore, the study in this paper provides a practical example of base station antenna for the sub-6 GHz bands in 5G mobile communications.

## Figures and Tables

**Table 1:** Performance comparison.

	Operating Frequency (GHz)	Bandwidth (GHz, %)	Minimum Port Isolation (dB)	Peak Gain (dBi)	HPBW (°)
[9]	3.65–3.81	0.16, 4.3% @RL > 10 dB	31	10.5	53.5 ± 1.5
[10]	3.4–3.8	0.4, 11.1% @RL > 15 dB	11.5	4.1	-
[11]	3.2–3.9	0.7, 19.7% @RL > 15 dB	25	7.9	69 ± 1
[12]	2.84–5.17	2.33, 58.3% @RL > 10 dB	-	6.2	-
Proposed antenna	3.14–5.04	1.9, 46.5% @RL > 15 dB	32.5	8.6	71.8 ± 2.5

Note: “RL” is return loss, and “-” means not provided.

**Table 2:** Geometrical parameters of the proposed antenna.

Parameter	Value (mm)	Parameter	Value (mm)
La	12.8	Hs	18
Ta	2.0	Ts	1.0
Ra	2.7	Lc	8.5
Ga	1.5	Wc	1.5
Tp	0.2	Tc	0.3
Rp	2.0	Gc	0.5
Ws	3.9	Wc1	1.0

**Table 3:** Half power beam widths (HPBWs) of the proposed antenna.

H-Plane		V-Plane	
Frequency (GHz)	HPBW (°)	Frequency (GHz)	HPBW (°)
3.3	70.5	3.3	70.9
4.15	69.3	4.15	71.5
5.0	73.2	5.0	74.3
